# Advances in the Diagnosis and Treatment of Acute Kidney Injury in Cirrhosis Patients

**DOI:** 10.1155/2017/8523649

**Published:** 2017-05-10

**Authors:** Lei Lei, Liangping Li, Hu Zhang

**Affiliations:** ^1^Department of Gastroenterology and Hepatology, West China Hospital, Sichuan University, Chengdu, China; ^2^Department of Gastroenterology and Hepatology, Sichuan Provincial People's Hospital, Chengdu, China

## Abstract

Liver cirrhosis is a common progressive and chronic clinical liver disease. Due to the strong compensation ability of the liver, no obvious symptoms develop in the early stage. However, multiple systems are involved in decompensation of the liver. Acute kidney injury (AKI) is one of the most serious complications, characterized by a sharp drop in the glomerular filtration rate (GFR); a rapid increase in Scr and BUN, as well as sodium and water storage; and a disturbance of acid-base balance. The mortality rate is high, and the prognosis is very poor. Thus, it is important to make a definite diagnosis and initiate treatment in the early stage to decrease mortality and improve the prognosis. Although diagnosing liver cirrhosis with serum creatinine has many shortcomings, a dynamic change in this marker is still the main diagnostic criterion for AKI. Identifying new markers of kidney injury with clinical value has also become an increasing focus of research. In this text, we review recent changes regarding categorization of AKI diagnostic criteria as well as new markers of AKI and treatments for cirrhosis-related AKI.

## 1. Background

Cirrhosis is a common clinical liver disease that is progressive and chronic. Due to the strong compensation ability of the liver, no apparent symptoms develop in the early stage. In contrast, multiple systems are affected in the decompensation stage. Acute kidney injury (AKI) is one of the most serious complications, especially in end-stage liver disease. AKI is characterized by a sharp drop in the glomerular filtration rate (GFR), a rapid increase in Scr and BUN, and increased sodium and water storage.

The etiology of cirrhosis-related AKI is as follows: (1) hypovolemia: an absolute shortage of blood volume, observed in conditions such as hemorrhage, diarrhea, excessive diuresis, and large-volume paracentesis; in contrast, a relative shortage of blood volume results from severe and unique cirrhosis-related abnormities of hemodynamics and nondiuretic, antihypertensive drugs; (2) inflammation: sepsis, including spontaneous bacterial peritonitis (SBP); (3) severe systemic response syndrome, which has separate causes; and (4) use of nephrotoxic drugs such as nonsteroidal anti-inflammatory drugs (NSAIDS), aminoglycosides, and radiographic contrast agents [1].

AKI develops in approximately 19% of hospitalized patients with cirrhosis [2]. It is a key predictive parameter for prognosis [3], suggesting a very poor result for patients with cirrhosis. It is estimated that AKI can increase the likelihood of death at day 30 by almost 10-fold in patients with cirrhosis [4]. Therefore, it is important to make a definitive diagnosis in the early stage and to prescribe appropriate medications to avoid mortality and improve prognosis. It is also necessary to improve our knowledge and understanding of AKI and cirrhosis-related AKI.

## 2. Diagnostic Criteria for AKI

AKI diagnosis is controversial due to a lack of unified diagnostic criteria [5], although some criteria, such as the RIFLE criteria, AKIN criteria, and KDIGO criteria, have been published. The Acute Dialysis Quality Initiative (ADQI) group first proposed the RIFLE diagnostic criteria in 2004. On the basis of the RIFLE criteria, the Acute Kidney Injury Network (AKIN) criteria were established in 2007. Partly based on the AKIN and RIFLE criteria, Kidney Disease: Improving Global Outcomes (KDIGO) published the KDIGO standard for the evaluation and management of AKI in 2012.

*RIFLE criteria* include parameters present during the whole course of the condition, ranging from kidney injury to end-stage renal failure. The criteria divide AKI into three levels, namely, risk, injury, and failure, according to changes in Scr, GFR, and urine volume. The prognosis of AKI is classified into two levels, namely, loss of renal function and end-stage renal disease (ESRD), based on the time of complete loss of renal function [5]. The RIFLE criteria have good maneuverability, high sensitivity, and high specificity in clinical research and can predict the prognosis of cirrhosis patients with AKI to a certain extent [6]. However, the criteria have some weaknesses; for example, Scr plays the same role as change in urine volume in assessing renal function, and GFR measurement is instable. Given these limitations, AKIN modified the RIFLE criteria and created its own criteria in 2007 to disseminate knowledge of AKI (Table 1).

*The AKIN criteria also classify* AKI into three stages, namely, dangerous, injury, and failure, but the parameter of GFR is excluded. In addition, the time window defining AKI development is limited to no longer than 48 h, and the threshold of Scr is set to no less than 26.5 *μ*mol/L, with or without a 50% increase from baseline within seven days. The change in the absolute value of Scr is emphasized here to indicate that a slight change in Scr could suggest a severely poor prognosis and that the baseline Scr level is a predictive parameter for renal function reversibility [7] (Table 1).

*The KDIGO criteria* were formulated on the basis of both the AKIN and RIFLE criteria. Some of the parameters drawn from the AKIN criteria include an increase in Scr ≥ 0.3 mg/dl (26.5 *μ*mol/L) or ≥50% baseline within 48 h and a urine volume < 0.5 mL/kg/h for more than 6 h. The parameters derived from the RIFLE criteria include an increase in Scr ≥ 50% baseline within 7 d or a decrease in GFR > 25% and a urine volume < 0.5 ml/kg/h for more than 6 h. The KDIGO criteria have the strengths of both the RIFLE and AKIN criteria by selectively including various parameters, but their reliability and sensitivity should be further tested in clinical studies [8] (Table 1).

## 3. Diagnosis of Cirrhosis-Related AKI

AKI has been widely recognized, but AKI in patients with cirrhosis is still a great challenge in clinical practice. The general diagnostic criteria for cirrhosis-related AKI are an increase in Scr ≥ 50% of baseline and >1.5 mg/dl (133 *μ*mol/l). To ensure early diagnosis and good management of AKI, the International Club of Ascites (ICA) created a new definition for cirrhosis-related AKI in 2015 [9] (Table 2).

Due to the inaccuracy of urine volume records for cirrhosis patients, a dynamic change in Scr is a key parameter to ensuring an accurate diagnosis [9]. The main differences between the new criteria and the general criteria for cirrhosis patients are as follows [9]. (1) An absolute value of Scr is highlighted. (2) The criterion for the cut-off value of Scr ≥1.5 mg/dl (133 *μ*mol/L) has been removed. (3) The staging system for AKI ensures a good assessment of both the progression stage and the regression stage because it allows a slightly longer time of one week to monitor a change in Scr. In the new ICA criteria for AKI, the urine output criterion has been removed because it is not appropriate for patients with cirrhosis (i.e., many patients with cirrhosis are oliguric but can maintain normal kidney function).

## 4. Categories of Cirrhosis-Related AKI

AKI can be divided into prerenal azotemia (PRA), acute tubular necrosis (ATN), and hepatorenal syndrome (HRS). Prerenal azotemia (PRA) results from various factors caused by the effective reduction of circulating blood volume. The reduction leads to a decrease in renal perfusion pressure. Consequently, the GFR cannot be maintained at a normal level, but renal tissue integrity is not damaged. If risk factors are removed at an early stage, renal function can be reversed to normal in most patients. Acute tubular necrosis (ATN) results from renal tubular epithelial cell injury/necrosis caused by renal ischemia and/or toxic damage, which leads to a dramatic decline in GFR, severe electrolyte imbalance, water sodium retention, and metabolic acidosis.

The HRS diagnostic criteria devised by the ICA in 1996 [10] are as follows: (1) Scr > 132.6 *μ*mol/L; (2) HRS caused by hypovolemia, ATN, use of nephrotoxic drugs, inflammation, or chronic kidney disease; and (3) HRS divided into type I and type II HRS according to the pace of deterioration. Type I HRS is a special form of AKI and is one of the most serious syndromes of cirrhosis decompensation and acute liver failure [11, 12]. HRS does not respond to fluid expansion. HRS thus has a poor prognosis, even if terlipressin, human albumin, and dialysis are used [12]. Type II HRS is characterized by a slow and progressive decline of renal function and mainly occurs in patients with refractory ascites. The treatment strategy for AKI varies between different types; thus, it is important to make the correct diagnosis. Differential diagnosis is difficult because the clinical characteristics of the two types are similar, and they can convert into one another or coexist.

The HRS criteria were revised in 2007 [13] as follows: (1) cirrhosis with ascites; (2) Scr > 132.6 *μ*mol/L; (3) no decrease in Scr (≤132.6 *μ*mol/L) 2 days after withdrawal of diuretics and expansion with albumin; (4) recommended albumin dosage of 1 g/(kg*∗*d) and maximum dosage of 100 g/d; (5) no shock history; (6) no recent use of nephrotoxic drugs; and (7) no albuminuria (>500 mg/d), no microscopic hematuria (urine RBC > 500/HP), and no renal parenchymal disease detected by ultrasonic examination. Further modification of the diagnostic criteria for HRS-related AKI was performed by the ICA in 2015. The use of Scr > 132.6 *μ*mol/L was removed, and AKI was defined as an absolute increase in Scr ≥ 0.3 mg/dL (26.5 *μ*mol/L) or ≥50% from baseline within 7 days.

## 5. Assessment of Renal Function

### 5.1. Traditional Markers Used to Assess Renal Function

*Scr* is the most practical and agreed upon biomarker for the assessment of renal function in cirrhosis patients [14], and it is the primary marker with which all types of renal failure can be predicted. However, there are some limitations to using Scr; namely, it may be normal or slightly increased because of high compensation and renal tubular secretion of creatinine in the presence of apparent kidney injury. These factors can lead to a delay in obtaining the correct diagnosis and initiating early management. Malnutrition exists in 67% of patients with cirrhosis, and production of creatinine from creatine decreases in muscles secondary to muscle wasting; therefore, Scr may be normal even if GFR is very low. The ability of this marker to assess renal function is much poorer. It can be influenced by some nonkidney factors, such as age, gender, race, prerenal factors, metabolism, and nutrition. The lab value of Scr may be lower than its actual value in patients with hyperbilirubinemia [15]. Furthermore, Scr cannot reveal the cause of AKI, so it is not a sufficiently sensitive marker to assess cirrhosis with AKI at an early stage and is therefore not ideal.

*GFR* is currently the best indicator of renal function. Clinically, MDRD and the Cockcroft Gault formula are used to assess GFR in the general population. Nevertheless, both overestimate GFR in cirrhosis patients [14]. Furthermore, although MDRD has more advantages regarding its use in the assessment of GFR in cirrhosis patients, its accuracy is much lower than that in noncirrhosis patients. The Cockcroft Gault formula is greatly influenced by weight, so it is not used for cirrhosis patients with edema and ascites [16].

*Urine volume* is a key marker for assessing kidney injury [14]. However, it is controversial to consider urine volume in patients with decompensated liver cirrhosis. Urine volume is affected by many factors, and its specificity is not high. For example, urine volume is normal in patients with nonoliguric AKI, despite the fact that their kidneys are severely damaged. Thus, urine volume has not been suggested for inclusion in the new ICA criteria for AKI diagnosis.

### 5.2. Emerging Markers to Assess Renal Function

*Scr is* still one of the main diagnostic criteria for AKI, although it has some disadvantages [9]. In particular, a dynamic change in Scr is a key criterion for cirrhosis-related AKI patients. The treatment strategy significantly differs among cases of AKI with different causes, so identification of the causal factors for AKI, while challenging, is very important. New biomarkers of kidney injury can distinguish structural AKI from functional AKI, which is very helpful for making a quick and accurate diagnosis. Several new markers have become topics of research, with studies mainly focused on CysC (Cystatin c), KIM-1 (kidney injury molecule 1), and NGAL (neutrophil gelatinase associated lipocalin). Reports from Europe and the United States have revealed that the combined application of NEGL or urine biomarkers [9], such as NGAL, KIM-1, and proteinuria, is potentially helpful for the differential diagnosis of cirrhosis-related AKI, but this should be further explored.

*CysC* is eliminated only through the kidney, and minor kidney damage could lead to a change in CysC [17]. Serum CysC concentration is mainly determined by GFR. If the GFR decreases by 20%, CysC will increase, so CysC is a reflection of early changes in ideal endogenous markers of GFR. A growing number of reports have demonstrated that CysC can be used as a marker for AKI assessment and prognosis [17]. Furthermore, CysC is not typically affected by age, gender, race, or weight; more specifically, it cannot be disturbed by hyperbilirubinemia. The sensitivity and specificity are 66% and 86%, respectively, when CysC > 1.23 mg/L. It is also a good predictive indicator of short-term mortality.

*KIM-1* is a unique and sensitive biomarker for early kidney injury [18]. KIM-1 is a type I transmembrane glycoprotein that contains immunoglobulin and a mucin domain. KIM-1 is expressed at a very low level in normal kidney tissues. It can exhibit high expression in dedifferentiated and proliferative renal tubule epithelium after kidney injury, but it is not detectable in totally atrophic tubular epithelia. KIM-1 is related to early injury and restoration of renal tubular epithelia. It is a new, noninvasive, and sensitive marker for early diagnosis of AKI, which is more specific and less susceptible to other factors, such as urinary tract infection (only at the urine test level). However, the detection of KIM-1 has not been standardized, and its independent value as a predictor of severe AKI is unclear [18].

*NGAL* is a new member of the lipocalin family. It has been reported that cisplatin, which can lead to renal tubular necrosis after intraperitoneal injection at a high dosage, can quickly induce the expression of kidney NGAL and its secretion from renal tubular cells [19]. NGAL, which is expressed in injured renal tubules and can induce epithelial regeneration, enters the blood within 2 h after injury and is excreted through urine. A study of 132 cirrhosis patients by Barreto et al. revealed that [20] the urine NGAL level in AKI patients was significantly higher than that in patients without AKI, and the NGAL level in consecutive AKI patients was significantly higher than that in temporary AKI patients. Thus, NGAL could be used to distinguish HRS from renal failure caused by other factors. Verna et al. reported [21] that the sensitivity and specificity of nonprerenal AKI diagnosis were 88% and 85%, respectively, when the urine NGAL density was 110 ng/mL. NGAL could predict the irreversibility of kidney function injury individually, so it might also predict mortality (which is independent of other risk factors) [22]. However, NGAL can be affected by systemic inflammation, and it is difficult to detect urine NGAL in oliguric and anuric patients.

## 6. Prevention and Treatment of Cirrhosis-Related AKI

### 6.1. General Treatment

Based on the ICA-AKI diagnostic criteria proposed for AKI in 2015 [9], we recommend that patients with cirrhosis and ascites who are in initial ICA-AKI stage 1 be managed as soon as possible with the following measures: (1) drug chart review, including review of all medications, reduction or withdrawal of diuretic therapy, and withdrawal of all potentially nephrotoxic drugs, vasodilators, or NSAIDs; (2) plasma volume expansion in patients with clinically suspected hypovolemia; and (3) prompt recognition and early treatment of bacterial infections when diagnosed or strongly suspected.

### 6.2. Drug Treatment

When AKI is characterized by an initial ICA-AKI stage 2 or 3 or by progression of the initial stage despite general therapeutic measures, patients who meet all the other diagnostic criteria for HRS should be placed on vasoconstrictors and albumin [9], irrespective of the final value of Scr. Vasoconstrictors can ameliorate vasodilatation in HRS patients, improve effective arterial blood volume (EABV), and ameliorate renal vasoconstriction and renal blood flow. Frequently used vasoconstrictors include terlipressin, midodrine, and noradrenaline. Continuous infusion is not required for terlipressin, and it has a low incidence of adverse effects; these advantages make it the first choice among vasoconstrictor analogues. Albumin can be combined with a vasodilator and can expand blood volume. As recommended by the European Association for the Study of the Liver (ESAL) [23] in 2010, terlipressin in combination with albumin should be considered the first-line therapeutic agent for type 1 HRS. The aim of this therapy is to improve renal function sufficiently to decrease Scr to <133 *μ*mol/l. Terlipressin plus albumin is effective in 60–70% of patients with type 2 HRS. If serum creatinine does not decrease by at least 25% after 3 days, the dosage of terlipressin should be increased in a stepwise manner up to a maximum of 2 mg/4 h. For patients with a partial response (serum creatinine does not decrease to <133 *μ*mol/L) or for those with no reduction of serum creatinine, treatment should be discontinued within 14 days. Albumin at 1 g/kg per day up to a maximum of 100 g/day over 2 days is recommended for HRS patients, with a subsequent change to 20–40 g/d. Terlipressin (0.5–2.0 mg, iv, once every 4–6 days) is given in combination with albumin. If the Scr level does not decrease, the dosage should be increased every few days up to the maximum dosage of 12 mg/d without adverse effects. The longest course should be 14 days.

It is reported [24] that the higher the initial Scr, the lower the response to terlipressin. Terlipressin in combination with albumin should be considered early when the Scr of a cirrhosis patient is higher than baseline and meets the AKI diagnostic criteria. There is no need to wait until the Scr level is higher than the ULN (>1.5 mg/dl). All AKI patients regardless of progression stage should be placed on vasoconstrictors if there is no obvious evidence of ATN or other renal diseases [3].

*Transjugular intrahepatic portosystemic shunt (TIPS)* has been reported to improve renal function in patients with decompensated cirrhosis and can also decrease their Scr [13, 25]. TIPS can improve refractory ascites, variceal bleeding, refractory hepatic pleural effusion, hepatorenal syndrome, refractory ascites, and variceal hemorrhage, which are the appropriate indications. Zhang and Zhao [26] reported that Scr was improved 7 days after TIPS and decreased to a normal level after 90 days if the Scr baseline was no more than 2 mg/dl, but the posttherapy MELD score was not significantly different from the score before therapy. If the Scr at baseline was more than 2 mg/dl, the Scr and MELD score were significantly improved after TIPS. Nie et al. reported [27] that TIPS can improve Scr and has a good effect on hemostasis with a low incidence of complications in addition to favorable clinical effects and a high safety rating. However, in clinical practice, attention should be paid to the following contraindications: Child-Pugh > 11 points, severe liver failure serum bilirubin > 5 mg/dl, severe cardiopulmonary dysfunction, severe coagulopathy, uncontrolled intrahepatic or systemic infection, biliary obstruction, portal vein cavernous transformation, and polycystic liver [16].

*Renal replacement therapy (RRT)* is important for AKI patients with decompensated cirrhosis. It can improve short-term survival and provide a basis for liver transplantation. Zhang et al. [28] reported on 284 severe AKI patients who were enrolled and received consecutive RRT. Renal function was recovered in 89 cases (31.33%). The incidence of chronic kidney disease in patients whose renal function was recovered was lower than that in patients whose renal function was not recovered. Moreover, the APACHE II score and organ failure number were relatively lower in patients whose renal function was recovered. These data suggest that complications, APACHE II score, and organ failure number are the key factors in RRT for AKI patients. In practice, the status of illness should be addressed in real time and RRT should be prescribed for patients as soon as possible, which can accelerate renal function recovery and improve survival.

*Liver transplantation* is one of the most important treatment strategies to improve the prognosis of ATN, which has the effect of improving survival and quality of life [24, 29]. Scr level before transplantation is an important predictive factor for mortality or renal dysfunction after surgery [30]. Therefore, renal function should be improved before surgery to improve outcomes and prevent renal failure [30, 31]. The incidence of renal failure after liver transplantation has decreased to 20% over time [32]. Liver transplantation for decompensated cirrhosis patients with AKI has been given increasing attention.

## 7. Conclusion

Cirrhosis-related AKI is caused by many factors and has high morbidity and mortality rates, so identifying the key causal factors is critical. The diagnostic criteria for cirrhosis-related AKI proposed by the ICA are the preferred choice for diagnosing AKI in cirrhosis. The assessment of renal function should be completed with traditional and emerging markers. A dynamic change in Scr is one of the most important diagnostic criteria, although it has some limitations. The exploration of new diagnostic markers has become a popular focus of research. Some treatments are currently available, such as removal of incentives, drug therapy, TIPS, and RPT. Liver transplantation is a good choice for refractory patients. It is imperative to make an early diagnosis and provide appropriate treatment for these patients to achieve a better outcome. A multicenter, prospective study with a large cohort of cirrhosis-related AKI patients that uses uniform criteria is warranted to elucidate the key causes of AKI and to develop better individual prevention and treatment strategies.

## Figures and Tables

**Table 1:** Current diagnostic criteria for acute kidney injury (AKI).

Criteria	Diagnostic criteria	Staging
*RIFLE* criteria	Increase in Scr to ≥1.5 times baseline within 7 days; GFR decrease >25%; or urine volume <0.5 ml/kg/h for 6 h	*Risk*. Scr increase of 1.5–1.9 times baseline; GFR decrease of 25–50%; or urine output < 0.5 ml/kg/h for 6 h *Injury*. Scr increase of 2.0–2.9 times baseline; GFR decrease of 50–75%; or urine output < 0.5 ml/kg/h for 12 h *Failure*. Scr increase ≥3.0 times baseline; GFR decrease of 50–75%; Scr increase ≥4.0 mg/dl (353.6 *μ*mol/L) with an acute increase of at least 0.5 mg/dl (44 *µ*mol/L); urine output < 0.3 ml/kg/h for ≥24 h; or anuria for ≥12 h

*AKIN* criteria	Increase in Scr by ≥0.3 mg/dl (26.5 *µ*mol/L) within 48 h; increase in Scr ≥ 1.5 times baseline within 48 h; or urine volume < 0.5 ml/kg/h for 6 h	*Stage 1*. Scr increase of 1.5–1.9 times baseline; Scr increase ≥0.3 mg/dl (26.5 *µ*mol/L); or urine output <0.5 ml/kg/h for 6 h *Stage 2*. Scr increase of 2.0–2.9 times baseline or urine output <0.5 ml/kg/h for 12 h *Stage 3*. Scr increase of 3.0 times baseline; Scr increase ≥4.0 mg/dl (353.6 *µ*mol/L) with an acute increase of at least 0.5 mg/dl (44 *µ*mol/L); urine output < 0.3 ml/kg/h for ≥24 h; or anuria for ≥12 h

*KDIGO* criteria	Increase in Scr by 0.3 mg/dl (26.5 *µ*mol/L) within 48 h; increase in Scr to ≥1.5 times baseline that is known or presumed to have occurred within the previous 7 days; or urine volume < 0.5 ml/kg/h for 6 h	*Stage 1*. Scr increase of 1.5–1.9 times baseline; Scr increase ≥0.3 mg/dl (26.5 *µ*mol/L); or urine output <0.5 ml/kg/h for 6–12 h *Stage 2*. Scr increase of 2.0–2.9 times baseline or urine output < 0.5 ml/kg/h for ≥12 h *Stage 3*. Scr increase of 3.0 times baseline; Scr increase to ≥4.0 mg/dl (353.6 *µ*mol/L); initiation of renal replacement therapy; urine output < 0.3 ml/kg/h for ≥24 h; or anuria for ≥12 h

**Table 2:** International Club of Ascites (ICA-AKI) 2015 definition for the diagnosis and management of AKI in patients with cirrhosis.

Subject	Definition
Baseline Scr	A Scr value obtained in the previous 3 months, when available, can be used as the baseline Scr. In patients with more than one value within the previous 3 months, the value closest to the admission time to the hospital should be used. In patients without a previous Scr value, the Scr upon admission should be used as the baseline value

Definition of AKI	Increase in Scr ≥ 0.3 mg/dl (≥26.5 *µ*mol/L) within 48 h or a percentage increase in Scr ≥ 50% from baseline that is known or presumed to have occurred within the previous 7 days

Staging of AKI	*Stage 1*. Increase in Scr ≥ 0.3 mg/dl (26.5 *µ*mol/L) or an increase in Scr ≥ 1.5-fold to 2-fold from baseline *Stage 2*. Increase in Scr > 2-fold to 3-fold from baseline *Stage 3*. Increase in Scr > 3-fold from baseline or Scr ≥ 4.0 mg/dl (353.6 *µ*mol/L) with an acute increase ≥0.3 mg/dl (26.5 *µ*mol/L) or initiation of renal replacement therapy

Progression of AKI	*Progression*. Progression of AKI to a higher stage or need for RRT *Regression*. Regression of AKI to a lower stage

Response to treatment	*No Response*. No regression of AKI *Partial Response*. Regression of AKI stage with a reduction of Scr to ≥0.3 mg/dl (26.5 *µ*mol/L) above the baseline value *Full Response*. Return of Scr to a value within 0.3 mg/dl (26.5 *µ*mol/L) of the baseline value
